# The association between frailty and hospital-related adverse events in older hospitalised patients: a systematic literature review

**DOI:** 10.1007/s41999-025-01242-8

**Published:** 2025-06-02

**Authors:** Faris Alotaibi, Abdullah Alshibani, Jay Banerjee, Brad Manktelow

**Affiliations:** 1 Department of Emergency Medical Care, College of Applied Medical Sciences, Imam Abdulrahman Bin Faisal University, Dammam, Saudi Arabia; 2 Department of Population Health Sciences, College of Life Sciences, University of Leicester, Leicester, UK; 3 Emergency Medical Services Department, College of Applied Medical Sciences, King Saud bin Abdulaziz University for Health Sciences, Riyadh, Saudi Arabia; 4 King Abdullah International Medical Research Center, Riyadh, Saudi Arabia; 5 Department of Emergency Medicine, University Hospitals of Leicester NHS Trust, Leicester, United Kingdom

**Keywords:** Frailty, Adverse event, Patient safety, Harms

## Abstract

**Introduction:**

Studies have shown that hospital-related adverse events (AEs) affect older hospitalised patients. Frailty is an age-related syndrome of increased vulnerability, the risk of abrupt and extreme health changes, and the risk of suffering an adverse event (AE). Most of the published work focuses on the relationship between age alone and AEs’ incidence. We aim to examine the association between frailty and the incidence of hospital-related AEs among hospitalised older adults in the published literature.

**Methods:**

A comprehensive search of Ovid MEDLINE, CINAHL, Scopus, and Web of Science databases was conducted between January and February 2024. Studies were included if they provide original data in English, regardless of methodology. In addition, the reference lists of all included studies were manually screened to identify any further eligible studies. The narrative synthesis followed the Cochrane methodology.

**Results:**

This review includes 19 observational studies, with the USA being the most common study location. Various frailty tools and definitions were used, with the frailty index (FI) being the most frequently employed tool. Hospital-acquired infections, including pneumonia, urinary tract infections (UTIs), sepsis and wound infections, were the most frequently reported adverse events, followed by delirium. In addition, falls, pressure ulcers and venous thromboembolism were also commonly documented.

**Conclusion:**

This review found that frailty is associated with an increased risk of hospital-acquired infections, in-hospital delirium, falls and pressure ulcers. More extensive and comprehensive studies are needed to focus on patient safety incidents among frail hospitalised individuals.

**Supplementary Information:**

The online version contains supplementary material available at 10.1007/s41999-025-01242-8.

## Key summary points

**Aim:**

Are hospitalised older patients living with frailty at different risk of hospital-related adverse events compared to non-frail patients?

**Findings:**

Frailty was associated with number of in-hospital adverse events, including hospital-acquired infections, delirium, in-hospital falls, pressure ulcers and venous thromboembolism.

**Message:**

More comprehensive studies are needed; frailty should be screened at admission of older patients, and adverse events reporting tools are encouraged to be embedded in healthcare system.

**Supplementary Information:**

The online version contains supplementary material available at 10.1007/s41999-025-01242-8.

## Introduction

In hospitals, adverse events (AEs) are a pressing problem for patients. Globally, 10% of hospitalised patients face one or more incidences of adverse events (AEs) while receiving care at a hospital [1]. A hospital-related AE refers to any unintended complication or negative outcome that arises during healthcare delivery which is not directly associated with the patient’s pre-existing medical condition or illness [2]. AE may produce a harm in various forms including, for example, increased in-hospital stay, disability on discharge or death [3]. The World Health Organization (WHO) further highlighted the most common AEs in its report *Patient Safety* [4]. These AEs include medication-related incidents, healthcare-associated infections, patient falls, pressure ulcers and venous thromboembolism.

The growing number of hospitalised older patients presents a significant challenge to the healthcare system in the UK. According to Age UK, older adults account for 41% of hospital admissions annually [5]. In addition, the National Audit Office (NAO) reported that individuals aged 65 years and older occupied 62% of hospital beds in 2016 [6]. Older patients are more likely to experience extended hospital stays, heightening their risk of hospital-related AEs during medical care [7, 8]. Prolonged length of stay (LOS) not only increases the likelihood of AEs but also contributes to negative health outcomes, such as the development of sarcopenia (loss of muscle mass) [6], and a higher risk of nosocomial infections (infections acquired in healthcare settings) [9]. Many older people are clinically living with frailty, putting them at increased risk of abrupt and extreme health changes, which increases their need for health and social care and increases the risk of long hospital stay if admitted [10].

Frailty is an age-related syndrome marked by increased vulnerability due to a decline in physiological function and homeostatic ability, leading to adverse health outcomes such as reduced physical activity, hospitalisation and higher mortality risk [11]. Older adults living with frailty face a high risk of prolonged hospital stays when admitted. Recent studies reveal that 50% of hospitalised older patients with frailty tend to remain in the hospital for more than 21 days [12]. Despite its significance, there is no globally accepted ‘gold standard’ for assessing frailty [13]. Instead, methodological approaches for assessing and classifying frailty are diverse, each based on different theoretical models and definitions. Yet, the two main models are the phenotypic model which focuses on physical symptoms of frailty [11], and the cumulative deficits model where a greater number of health deficits indicates increased frailty [14].

While most of the published studies assess the association between age and adverse events, there remains a significant gap in the literature regarding the relationship between frailty and the incidence of adverse events in hospitalised older adults. Identifying frailty in older hospitalised patients is particularly valuable because it often indicates reduced physiological reserves and resilience. Frailty is strongly associated with a heightened risk of adverse events during hospital admissions such as falls, delirium, pressure ulcers, infections and unfavourable recovery trajectories primarily due to diminished capacity to cope with stressors (e.g. surgery and acute illness) [10, 15]. Numerous studies have examined the risk and nature of hospital-related AEs and their associated harms during hospital care [1, 16–22]. Most published research has primarily examined the relationship between age and adverse events (AEs), with limited attention to frailty as an additional risk factor. To our knowledge, no systematic review has comprehensively gathered, analysed and assessed the available evidence on the association between frailty and hospital-related AEs. This systematic review aims to evaluate the relationship between frailty and the risk of hospital-related AEs among hospitalised older adults compared to their non-frail counterparts.

## Methods

### Protocol and registration

This systematic literature review followed the PRISMA (Preferred Reporting Items for Systematic Reviews and Meta-Analyses) guidelines and checklist [23]. The review protocol was registered with the International Prospective Register of Ongoing Systematic Reviews (PROSPERO: CRD42024560071) [24]. A detailed protocol for this systematic review was published in *BMJ Open* and is available at [25].

### Research question development

We developed the review question using the Population, Intervention, Comparison and Outcome (PICO) format to guide the systematic review. The population of interest consists of patients aged 65 years and older living with frailty. The intervention focussed on hospitalisation. Older adults with frailty were compared to non-frail older adults. The outcome of interest was hospital AEs. The research question was formulated as: are hospitalised older patients living with frailty at risk of hospital-related AEs?

### Search strategy

Two reviewers FA and AA generated, reviewed and developed a list of the main keywords derived from the PICO question and their alternatives and synonyms, including MeSH terms where applicable. Keywords were combined using the Boolean Operator “OR” to expand the search scope and “AND” to effectively integrate results from individual searches. See supplemental Table 1. An online data search was then performed using the listed keywords and their alternatives across four databases: MEDLINE, SCOPUS, CINAHL, and Web of Science. The search was conducted in January 2024 to February 31, 2024, focussing on peer-reviewed studies published in English.

**Table 1:** Quality assessment of the included studies

Studies	Q1	Q2	Q3	Q4	Q5	Q6	Q7	Q8	Q9	Q10	Quality assessment
Thillainadesan [26]	Y	Y	Y	Y	Y	Y	Y	Y	Y	Y	100% High
Esmaeeli [27]	Y	Y	Y	Y	Y	Y	Y	Y	Y	Y	100% High
Welch [28]	Y	Y	Y	Y	N	Y	Y	Y	Y	Y	90% High
Sieber [29]	Y	Y	Y	Y	N	Y	Y	Y	Y	Y	90% High
McEvoy [30]	Y	Y	Y	Y	N	Y	Y	Y	Y	Y	90% High
Hubbard [31]	Y	Y	Y	Y	N	Y	Y	Y	Y	Y	90% High
Joosten [32]	Y	Y	Y	Y	Y	Y	Y	Y	Y	Y	100% High
Hanlon [33]	Y	Y	Y	Y	N	N	Y	U	U	U	50% Moderate
Jung [34]	Y	Y	Y	Y	N	Y	Y	Y	Y	Y	90% High
Nowak [35]	Y	Y	Y	Y	N	Y	Y	Y	Y	Y	90% High
Kim [36]	Y	Y	Y	Y	N	Y	Y	N	N	Y	70% Moderate
Chan [37]	Y	Y	Y	Y	N	Y	Y	Y	Y	Y	90% High
Birkelbach [38]	Y	Y	Y	Y	N	Y	Y	Y	Y	Y	90% High
Chen [39]	Y	Y	Y	Y	N	Y	Y	Y	Y	Y	90% High
Aceto [40]	Y	Y	Y	Y	N	Y	Y	N	N	Y	70% Moderate
Dasgupta [41]	Y	Y	Y	Y	U	Y	Y	Y	Y	Y	90% High
Deiner [42]	Y	Y	Y	Y	N	Y	Y	Y	Y	Y	90% High
Joseph [43]	Y	Y	Y	Y	N	Y	Y	U	U	U	60% Moderate
Leung [44]	Y	Y	Y	Y	U	Y	Y	Y	Y	Y	90% High

### Eligibility criteria

We included studies that defined their population as individuals 65 years and older, used at least one validated assessment tool to measure frailty and referred to participants as “frail” without applying a formal frailty assessment tool (e.g. Clinical Frailty Scale, Frailty Index, and Fried Phenotype) were excluded. Furthermore, we included studies that reported any hospital-related AEs during hospitalisation. Only studies reporting original data and published in English were eligible. We excluded abstracts, editorials, protocols, letters to the editor, and correspondences. In addition, studies that followed patients post-discharge or reported AEs in outpatient, community-based or rehabilitation settings were excluded. Only studies conducted in acute hospital settings were included, and no geographic restrictions were applied.

### Study selection process

The studies identified from the four databases were extracted and uploaded to Rayyan AI, an artificial intelligence tool to help systematic reviews articles screenings [45]. Rayyan AI was used to facilitate title and abstract screening, as well as for facilitating the detection and removing duplicates. Two independent reviewers FA and AA carried out the screening and selection process, with a third reviewer JB available to resolve any disagreements that could not be settled through discussion. Initially, FA and AA screened titles and abstracts based on the inclusion and exclusion criteria. Then, a full-text screening of the remaining studies was conducted to determine which studies would be included in the systematic review. Finally, a hand search was performed on the reference lists of included studies to identify additional eligible studies. The final selection of studies was agreed upon by FA and AA and presented using the PRISMA flowchart.

### Risk of bias

The two independent reviewers FA and AA used a modified version of the Joanna Briggs Institute (JBI) Critical Appraisal Checklist for Analytical Cross-Sectional Studies to assess the risk of bias and the quality of all the included studies in the review [46]. See supplemental Table 2. The checklist consists of ten sections, each having the potential to be rated as “Yes”, “No”, “Unclear”, and “Not Applicable”. The quality assessment was calculated based on the proportion of questions answered “YES” only. Each study was then categorised based on the answers to the questions as either “Low Quality” <50%, “Moderate Quality” 50–70% or “High Quality” >70%. No study was excluded from this review based on the risk of bias.

**Table 2:** Characteristics of the included studies

Authors	Country	Year	Sample size	Age (mean or median)	Gender	Frailty tool	Type of AE
Deiner et al. [42]	USA	2023	505	76.7 (SD ± 5.22)	Female n = 297 (58.8%)	Frailty index and frailty phenotype	Postoperative delirium POD
Esmaeeli et al. [27]	USA	2022	556	85 (SD ± 7)	Female 388 (69.78%)	FRAIL Score	Postoperative delirium POD
Sieber et al. [29]	USA	2022	324	4AT < 4 = mean age 73.1 (SD 6.2)/4AT ≥ 4 = mean age 77.5 (SD 6.3)	Female 128 (39.5%)	Edmonton frailty scale EFS	Postoperative delirium POD
Joseph et al. [43]	USA	2017	350	Non-frail: 76.5/SD = 5.1 Pre-frail: 76.9/SD = 7.9 Frail: 77.5/SD = 8.4	Non-frail (n = 126): 63 males (50%), 63 females (50%) Pre-frail (n = 91): 62 males (68%), 29 females (32%) Frail (n = 133): 71 males (53%), 62 females (47%)	Trauma-Specific Frailty Index (TSFI)	Urinary tract infection UTI, pneumonia, sepsis, disseminated intravascular coagulation DIC, deep venous thrombosis DVT and pulmonary embolism PE
Leung et al. [44]	USA	2010	63	Delirium 74.2 SD 6.0 No delirium 71.9 SD 6.3	Female: Delirium (n = 16) 8 (50%) No delirium (n = 47) 26 (55%)	Fried phenotype	Postoperative delirium POD
Hanlon et al. [33]	USA	2004	397	Aged 65–74/213 (53.6%), Aged ≥ 75/184 (46.4%)	Female 11 (2.8%)	10-item frailty score based on Veterans Affairs (VA) Cooperative Study health service trial	Inappropriate drug prescribing
Mcevoy et al. [30]	Australia	2023	44,721	80 (SD 7)	Female 25,306 (57%)	Clegg’s clinical ICD-10-AM coded cumulative deficit items	Falls, pressure injury, delirium, pneumonia, thromboembolism
Thillainadesan et al. [26]	Australia	2021	150	79.5 (SD ± 7.7)	Males 102 (68.0%)	The clinical frailty scale CFS and a 37-item FI based on the Rockwood FI	Functional decline, constipation, pressure injury, fall, delirium
Hubbard et al. [31]	Australia	2017	1418	81.0 (SD 6.8)	Female 780 (55.0%)	A frailty index (FI-AC)	Inpatient fall, delirium, pressure ulcer functional decline
Chan et al. [37]	Canada	2019	423	CFS 1–3 n = 71 mean SD: 77.1 (6.9)/CFS 4 n = 72 mean SD: 79.3 (8.1)/CFS 5 n = 92 mean SD: 83.4 (7.2)/CFS 6–9 n = 187 mean SD: 85.2 (8.2)	Female: CFS 1–3 n = 45 (63.4%)/CFS 4 n = 47 (65.3%)/CFS 5 n = 50 (54.4%)/CFS 6–9 n = 125 (66.8%)	Clinical Frailty Scale CFS	Delirium, venous thromboembolism, pneumonia, urinary tract infection, myocardial injury, need for transfusion, pressure ulcer and falls
Dasgupta et al. [41]	Canada	2009	125	Entire cohort (mean): 77.4/Patients without complications (n = 94): 76.3 ± 5.0/Patients with complications (n = 31): 80.8 ± 5.8	Female: n = 72 (58%)	Edmonton frailty scale EFS	Cardiac complications: one of either ischaemia, congestive heart failure, new arrhythmia or sudden death Pulmonary complications: one of either pneumonia, significant bronchospasm, deep venous thrombosis or pulmonary embolisms (DVT or PE), the excessive need for respiratory support. Delirium
Jung et al. [34]	South Korea	2022	1016	CFS < 5 (n = 637) 71.8 ± 5.1, CFS ≥ 5 (n = 379) 75.0 ± 7.2	Female CFS < 5 (n = 637) 215 (33.8%), CFS ≥ 5 (n = 379) 200 (52.8%)	Clinical frailty scale CFS	Fall, pressure ulcer, delirium
Kim et al. [36]	South Korea	2021	85	74.05(SD ± 6.47)	Female 50 (58.8%)	Korean version of the fatigue, resistance, ambulation, illnesses, and loss of weight (K-FRAIL) scale	Poor oral intake, Voiding difficulty, urinary tract infection, delirium, pneumonia, postoperative hematoma, acute kidney injury, electrolyte imbalance, sepsis, cardiac arrest, superficial wound infection
Nowak et al. [35]	Poland	2023	174	Robust 70.8 ± 4.2, Pre-frail 72.8 ± 5.8, Frail 79.2 ± 8.0	Female: Robust (n = 54) 15 (27.8%) Pre-Frail (n = 52) 24 (46.1%), Frail (n = 68) 39 (57.4%)	FRAIL Score	Bleeding, infection, arrhythmia, acute kidney injury (AKI), delirium, stroke/transient ischaemic attack (TIA), liver injury, hypoglycaemia
Chen et al. [39]	China	2022	227	71.2 [SD ± 4.8]	Male 140 (61.7%)	FRAIL Score	Postoperative pulmonary complications PPCs (pneumonia, pulmonary congestion, bronchospasm, atelectasis, pneumothorax, respiratory failure, pleural effusion or requirement for mechanical ventilation)
Aceto et al. [40]	Italy	2021	97	With PPC 73.3 ± 7.1/Without PPC 69.7 ± 4.7	Female n = 47 (44.7%)	Modified Frailty Index (mFI)	PPCs (respiratory failure, pulmonary infection, aspiration pneumonia, pleural effusion, pneumothorax, atelectasis on chest X-ray, bronchospasm, or un-planned urgent re-intubation)
Welch et al. [28]	UK	2019	1507	80.0 (SD ± 8.3)	Female 798 (54.2%)	Clinical frailty scale CFS	Delirium
Birkelbach et al. [38]	Germany	2019	1186	74.0 [SD ± 4]	Male 623 (52.5%)	Fried phenotype	Pneumonia, pulmonary embolism, acute kidney injury, cerebrovascular accident, coma, superficial and deep wound infections, urinary tract infection, sepsis, deep vein thrombosis, myocardial infarction, cardiac arrest
Joosten et al. [32]	Belgium	2014	220	Pre-frail and non-frail 83.7 ± 4.8, Frail 83.3 SD ± 5.4)	Female 126 (57%)	Cardiovascular Health Study (CHS) and the Study of Osteoporotic Fracture (SOF) frailty index	In-hospital delirium and falls

### Data extraction, synthesis and analysis

Data were extracted from all included studies using a data extraction form developed by the study team on an MS Excel sheet, including title, inclusion decision, reason of decision, year, author, place of the study, study aim, study design, population included in the study, sample size, age (mean or median), gender, frailty tools used, definition of frailty, type of AE, incidence of AE n (%), frailty and AE association, effect estimates, preventability of measured AE and authors’ conclusion. FA and AA independently extracted data with the revision of two expert reviewers JB and BM. Meta-analysis was not feasible due to the heterogeneity of frailty-measuring tools that use diverse frailty classifications. In addition, the included studies measure different AEs and different effect estimates. Therefore, a narrative synthesis was performed based on Cochrane guidelines for conducting systematic reviews [47]. Studies that met the inclusion criteria were synthesised based on frailty measuring tool, types of AEs and the association between frailty and AE. Given the variability in the types of AEs reported across studies, we prioritised AEs with a clear potential to cause harm to patients during hospital care. This included complications such as infections, delirium, falls and pressure ulcers. Studies reporting broader AE outcomes were also included if the events were clinically relevant and occurred during the hospital stay.

## Results

**Fig. 1:**
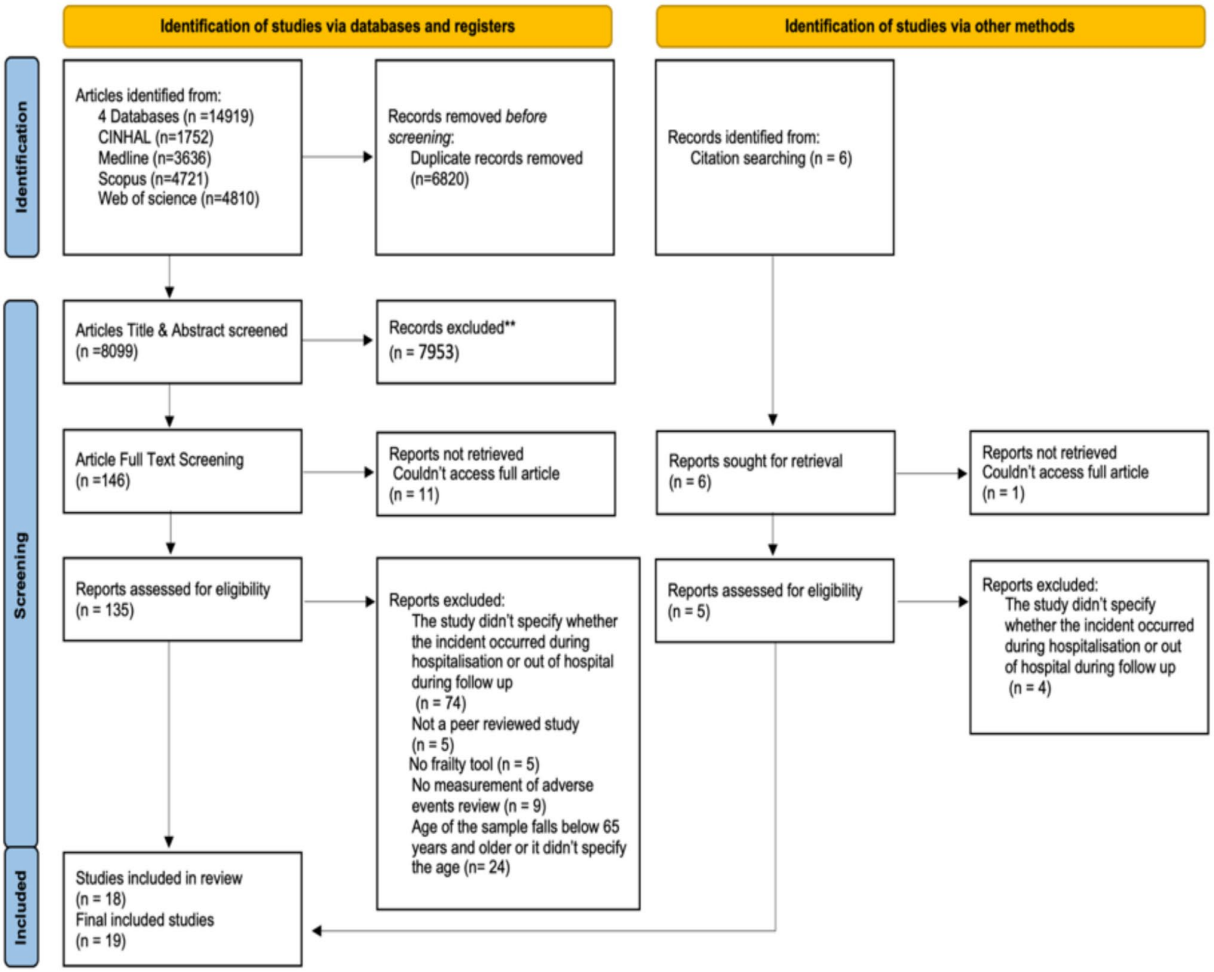
The modified PRISMA 2020 flow diagram for new systematic reviews, which included searches of databases and other sources. Abbreviation: PRISMA, Preferred Reporting Items of Systematic Reviews and Meta-Analysis [48]

The initial database search resulted in 14,919 studies; of these, 6,820 were found to be duplicates and removed. 8,099 studies progressed to title and abstract screening. The stage of title and abstract screening resulted in 146 possible studies that meet the inclusion criteria of the review. Of these, 11 were not available in full text and, therefore, were excluded. As a result, 135 studies progressed to full article screening. At this stage, 18 studies met the inclusion criteria and were included. One study was included from the reference list of the final included studies, resulting in 19 studies to be included in this review. See Figure 1.

### Risk of bias

Out of the 19 included studies, four were deemed moderate quality [33, 36, 40, 43]. The other 15 studies were found to be of high quality [26–32, 34, 35, 37–39, 41, 42, 44]. See Table 1.

### Studies characteristics

The characteristics of the 19 included studies in this review are listed in Table 2. All studies included in this review were observational studies; nine studies retrospectively collected data, and the other ten studies used a prospective approach to recruit participants. This review’s largest study population article was McEvoy’s study [30] with more than 44,000 included patients, compared to other included studies with a relatively small sample size. Six studies were conducted in the USA [27, 29, 33, 42–44], three in Australia [26, 30, 31], two studies in Canada [37, 41] and two in South Korea [34, 36], while the rest were conducted in the UK [28], China [39], Germany [38], Belgium [32], Italy [40] and Poland [35]. See supplemental Table 3.

### Frailty tools

All studies included in this review applied a recognised assessment tool to measure frailty, though the specific tools varied across most of the studies. Frailty was measured using a frailty index (FI) in four studies [31, 40, 43], Rockwood’s Clinical Frailty Scale (CFS) in three studies [28, 34, 37], the FRAIL Score in three studies [27, 35, 39], two studies applied the Edmonton Frail Scale (EFS) [29, 41], two used the Fried’s phenotype model [38, 44] and one study used the K-FRAIL scale which is a translated version of the FRAIL score to Korean language [36]. The rest of the studies used Clegg’s 34-item cumulative deficits model [30], and a 10-item frailty score based on Veterans Affairs (VA) Cooperative Study health service trial [33]. Some studies used a combination of two frailty tools: two studies used a FI and a frailty phenotype model [32, 42], and another used CFS and the 37-item FI based on the Rockwood FI [26].

### Types and preventability of in-hospital AEs

The most frequently measured and consistently reported adverse event (AE) across the included studies was hospital-acquired infections. Pneumonia was the most common, reported in nine studies [26, 30, 35–41, 43], followed by urinary tract infections (UTI) in four studies [36–38, 43], sepsis in three studies [36, 38, 43] and wound infections in two studies [36, 38]. General infection [35] and pulmonary infection [40] each was reported in a single study. In-hospital delirium was another significant AE, identified in 14 studies [26–32, 34–37, 41, 42, 44], while in-hospital falls were noted in six studies [26, 30–32, 34, 37]. Pressure injuries were reported in five studies [26, 30, 31, 34, 37]. Venous thromboembolism was reported in five studies [30, 37, 38, 41, 43]. Less commonly, acute kidney injury was reported in three studies [35, 36, 38], functional decline in two [26, 31] and inappropriate drug prescription in one study [33]. Notably, only four studies highlighted the importance of identifying frailty as a key factor in preventing AEs by enabling tailored care strategies for this population [34, 35, 38, 43].

### Frailty association with the incidence of in-hospital AEs

A summary of the findings from the included studies is presented in Table 3. Among these, 17 studies reported an association, relationship, or predictive value of frailty measured using various tools with a range of in-hospital adverse events (AEs). One study, conducted by Joosten et al. [32] found that frailty, as assessed by the Cardiovascular Health Study (CHS) criteria and the Study of Osteoporotic Fractures (SOF) frailty index, had limited value in predicting falls and delirium among hospitalised older patients after adjusting for age and other confounders. However, the study did identify a significant association between frailty and 6-month mortality. Another study by Hanlon J et al. [33], which included only frail patients, found out that (91.9%) frail patients received ≥ 1 inappropriate medication prescribed. However, it did not use frailty as a predictor for the outcome in this study.

**Table 3:** The association between frailty and hospital-related adverse events

Authors	Type and incidence of AE n (%) Among non-frail and pre-frail patients (if reported)	Type and incidence of AE n (%) Among frail patients	Adjusted effect estimates for frailty as a predictor of AE	Confounders
Deiner et al. [42]	Postoperative delirium (POD) Frailty index (FI): Non-frail 17/120 Pre-frail 32/190 Frailty phenotype (FP): Non-frail 8/64 Pre-frail 58/280	Postoperative delirium (POD) Frailty index (FI): Frail: 60/195 Frailty phenotype (FP): Frail: 43/160	Frailty index RR 1.84 [95% CI 1.05–3.21], *p*-value = 0.06 Frailty phenotype RR 1.33 [95% CI 0.63–2.80], *p*-value = 0.78	Adjusted for: age, sex, ASA class, surgical urgency, CCI, procedure complexity, and Modified Mini-Mental State
Esmaeeli et al. [27]	Postoperative delirium (POD) Robust (frail 0): 7/174 Pre-frail (frail 1–2): 36/232	Postoperative delirium (POD) Frail (frail 3–5): 37/151	OR 1.33 (95% CI 1.02–1.72), *p*-value = 0.03	Adjusted for: age, sex, marital status, cognitive score, Charlson comorbidity Index, falls in the past year, glomerular filtration rate, and ICU admission
Sieber et al. [29]	Postoperative delirium (POD) Non-frail: 5/241	Postoperative delirium (POD) Frail: 10/83	OR 3.5 (95% CI 1.1–11.5), *p*-value = 0.0007	Adjusted for: age and comorbidities
Joseph et al. [43]	Non-Frail and pre-frail UTI: 9/217 Pneumonia: 7/217 Sepsis: 6/217 DVT/PE: 5/217 DIC: 2/217	Frail UTI: 13/133 Pneumonia: 12/133 Sepsis: 5/133 DVT/PE: 7/133 DIC: 2/133	Not reported	Not reported
Leung et al. [44]	Postoperative delirium (POD) Pre-frail (frailty scores 1–2): 7/32	Postoperative delirium (POD) Frail (frailty score ≥ 3): 9/21	OR 1.84 (95% CI 1.07–3.15), with a *p*-value of 0.028	Adjusted for: age, GDS score, and baseline cognitive function
Hanlon et al. [33]	The population of this study consisted of all frail individuals, with 365/397 (91.9%) frail patients receiving ≥ 1 inappropriate medication prescribed		Not reported	Not reported
McEvoy et al. [30]	Falls 0–1 deficit: 104/24,339 2 deficits: 181/8,717 3 deficits: 318/8,513 Pressure injury 0–1 deficit: 19/24,339 2 deficits: 17//8,717 3 deficits: 50//8,513 Delirium 0–1 deficit: 63/24,339 2 deficits: 202/8717 3 deficits: 426/8513 Pneumonia 0–1 deficit: 88/24,339 2 deficits: 104/8717 3 deficits: 219/8513 Thromboembolism 0–1 deficit: 33/24,339 2 deficits: 25/8717 3 deficits: 59/8513	4–12 deficits Falls: 217/3152 Pressure injury: 54/3152 Delirium: 351/3152 Pneumonia: 189/3152 Thromboembolism: 31/3152	4–12 deficits Falls: RR 15.33 (95% CI 12.1–19.42, *p* < 0.001) Pressure ulcers: RR 21.28 (95% CI 12.53–36.16, *p* < 0.001) Delirium: RR 40.88 (95% CI 31.21–52.55, *p* < 0.001) Pneumonia: RR 16.46 (95% CI 12.74–21.27, *p* < 0.001) Thromboembolism: RR 7.25 (95% CI 4.4–11.92, *p* < 0.001)	Adjusted for: age and sex
Thillainadesan J et al. [26]	Assessed by a FI Delirium: 6/116 Functional decline: 17/116 Constipation: 64/116 Fall: 3/116 Pressure injury: 2/116 Assessed by CFS Delirium: 6/105 Functional decline: 15/105 Constipation: 59/105 Fall: 3/105 Pressure injury: 2/105	Assessed by a FI Delirium: 9/34 Functional decline: 11/34 Constipation: 16/34 Fall: 3/34 Pressure injury: 0/34 Assessed by CFS Delirium: 10/45 Functional decline: 13/45 Constipation: 21/45 Fall: 3/45 Pressure injury: 0/45	Frailty Index (FI): Delirium: OR = 5.64 (95% CI 1.47–21.68) Functional decline: OR = 2.08 (95% CI 0.73–5.91) Clinical frailty scale (CFS): Delirium: OR = 4.21 (95% CI 1.14–15.50) Functional decline: OR = 1.39 (95% CI 0.50–3.88)	Adjusted for: age, sex, Charlson comorbidity index (CCI), admission type and surgical management approach
Hubbard et al. [31]	The study did not explicitly report the exact numbers of AE per frail vs non-frail patients Inpatient fall: 83/1418 Inpatient delirium: 321/1418 Inpatient pressure ulcer: 42/1418 Inpatient functional decline: 96/1418		Inpatient Falls: OR 1.29 (95% CI: 1.10–1.50) Per 0.1 FI increase. Delirium: OR 2.34 (95% CI: 2.08–2.63) Per 0.1 FI increase. Pressure Ulcers: OR 1.51 (95% CI: 1.23–1.87) Per 0.1 FI increase. Functional decline: OR 1.20 (95% CI: 1.04–1.40) Per 0.1 FI increase.	Adjusted for: age, gender, comorbidities, and baseline cognitive function
Chan et al. [37]	CFS 1–3 Delirium: 21 Venous thromboembolism: 1 Pneumonia: 2 UTI: 7 Myocardial injury: 11 Need for transfusion: 14 Pressure ulcer: 8 Fall: 1	CFS 4–9 Delirium: 240 Venous thromboembolism: 12 Pneumonia: 28 UTI: 49 Myocardial injury: 104 Need for transfusion: 121 Pressure ulcer: 116 Fall: 22	(CFS 4) OR 1.0 (95% CI 0.5–2.2), *p*-value: 0.974 (CFS 5) OR 2.1 (95% CI 0.9–4.8), *p*-value: 0.076 (CFS 6–9) OR: 4.8 (95% CI 2.1–10.8), *p*-value: < 0.001	Adjusted for: age, sex, time to surgery, and mode of anaesthesia
Dasgupta M et al. [41]	Having at least one complication: EFS < 4: 5/51 EFS 4–7: 48/183	Having at least one complication: EFS > 7: 9/16	EFS > 7 Cardiac complication: OR 3.75 (95% CI 1.04–13.51) Pulmonary complication: OR 6.61 (95% CI 1.51–28.29) Delirium: OR 2.43 (95% CI 0.65–9.07)	Adjusted for: age
Jung et al. [34]	For CFS < 5 Total non-frail patients = 637 Delirium 1/637 Pressure ulcers 0/637 Falls 1/637	For CFS ≥ 5 Total frail patients = 379 Delirium 49/379 Pressure ulcers 26/379 Falls 5/379	Falls: OR = 1.39 (95% CI 0.74–2.60) Pressure ulcers: OR = 2.77 (95% CI 1.94–3.96) Delirium: OR = 2.56 (95% CI 1.98–3.31)	Adjusted for: age and sex
Kim et al. [36]	The study reported the total incidence of adverse events but did not specify the breakdown between non-frail and frail groups Total incidence of complications = 85 Poor oral intake 16/85 Voiding difficulty 14/85 Urinary tract infection 4/85 Delirium 3/85 Pneumonia 3/85 Postoperative hematoma 2/85 Acute kidney injury 1/85 Electrolyte imbalance 1/85 Sepsis 1/85 Cardiac arrest 1/85 Superficialwoundinfection1/85 , *p*		K-FRAIL scale and any postoperative complication:OR = 0.130 (95% CI 0.039–0.222), p = 0.006	Adjusted for: age, surgical invasiveness, and CCI
Nowak et al. [35]	Non-frail: Bleeding 9/54 Infection 6/54 arrhythmia 5/54 Acute kidney injury (AKI) 11/54 Delirium 2/54 Stroke/transient ischaemic attack (TIA) 1/54 Liver injury 0/54 Hypoglycaemia 1/54 Pre-frail: Bleeding 14/52 Infection 16/52 Arrhythmia 19/52 Acute kidney injury (AKI) 15/52 Delirium 6/52 Stroke/transient ischaemic attack (TIA) 2/52 Liver injury 6/52 Hypoglycaemia 0/52	Frail: Bleeding 31/68 Infection 37/68 Arrhythmia 54/68 Acute kidney injury (AKI) 39/68 Delirium 36/68 Stroke/transient ischaemic attack (TIA) 3/68 Liver injury 12/68 Hypoglycaemia 7/68	Bleeding: not reported Infection: OR = 3.3 (95% CI 1.6–7.0) Pneumonia/LRTI: OR = 2.5 (95% CI 1.1–5.8) UTI: OR = 4.8 (95% CI 1.8–12.5) Arrhythmia (only atrial fibrillation): OR = 3.5 (95% CI 1.3–9.3) AKI: OR = 2.6 (95% CI 1.2–5.3) Delirium: OR = 11.7 (95% CI 4.8–28.7)	Adjusted for: age, BMI, atrial fibrillation, left ventricular ejection fraction, haemoglobin on admission, and invasive vs. conservative treatment
Chen et al. [39]	Postoperative pulmonary complications (PPC) = 56 Non-frail: 20/56	Postoperative pulmonary complications (PPC) = 56 Frail: 30/56	Frailty: OR 6.33 (95% CI 2.45–16.37)	Adjusted for: age, sex, body mass index, current drinker, current smoker, hypertension, diabetes, coronary artery disease, asthma, chronic obstructive pulmonary disease, obstructive sleep apnoea, bronchiectasis, respiratory infection within the last month, forced expiratory volume in the first second (FEV1), FEV1/forced vital capacity (FVC), preoperative SpO2, haemoglobin, creatinine, operation time
Aceto et al. [40]	Postoperative pulmonary complications (PPC) Non-frail: 1/11	Postoperative pulmonary complications (PPC) Frail: 10/11	Logistic regression showed that mFI were a predictor of PPCs (*p* = 0.0001) but the study did not report the OR	Not reported
Welch et al. [28]	Postoperative delirium (POD) Fit (CFS 1–3): 15 out of 468	Postoperative delirium (POD) Frail (CFS 4–6): 134/796 Very frail (CFS 7–9): 67/201	Frail (CFS 4–6): OR = 4.80 (95% CI 2.63–8.74) Very frail (CFS 7–9): OR = 9.33 (95% CI 4.79–18.17)	Adjusted for: age, sex, Charlson Comorbidity Index (CCI), admission type, and baseline cognitive status
Birkelbach et al. [38]	Non-frail and pre-frail Pneumonia: 23/1051 Pulmonary embolism: 6/1051 Acute kidney injury: 57/1051 Cerebrovascular accident: 2/1051 Coma: 2/1051 Superficial wound infections: 26/1051 Deep wound infections: 15/1051 Urinary tract infection: 172/1051 Sepsis: 14/1051 Deep vein thrombosis: 12/1051 Myocardial infarction: 2/1051 Cardiac arrest: 3/1051	Frail Pneumonia: 5/135 Pulmonary embolism: 0/135 Acute kidney injury: 12/135 Cerebrovascular accident: 1/135 Coma: 2/135 Superficial wound infections: 8/135 Deep wound infections: 3/135 Urinary tract infection: 33/135 Sepsis: 4/135 Deep vein thrombosis: 2/135 Myocardial infarction: 2/135 Cardiac arrest: 4/135	Frail OR 2.08 (95% CI 1.21–3.60) *p*-value, 0.008	Adjusted for: age, sex, body mass index, American Society of Anaesthesiologists Physical Status (ASA PS), surgical risk, type of anaesthesia, Charlson comorbidity index (CCI), surgical discipline, smoking status, polypharmacy, as well as preoperative creatinine levels and glomerular filtration rates (GFR)
Joosten et al. [32]	Non-frail and pre-frail by CHS Delirium: 14/132 Fall: 10/132 Non-frail and pre-frail by SOF Delirium: 14/138 Fall: 12/138	Frail by CHS Delirium: 10/88 Fall: 8/88 Frail by SOF Delirium: 6/66 Fall: 5/66	CHS index Delirium OR 0.64 (95% CI 0.25–2.08) Fall OR 0.94 (95% CI 0.31–2.91) SOF index Delirium OR 0.81 (95% CI 0.21–3.2) Fall OR 0.71 (95% CI 0.21–2.4)	Adjusted for: age, sex, education, number of comorbidities, ADLs, cognitive impairment, main diagnosis, haemoglobin, depression, and estimated Glomerular Filtration Rate

### Factors other than frailty impacting the incidence of AEs

Kim et al. [36] identified that comorbidities, measured by the Charlson Comorbidity Index (CCI), and surgical invasiveness, in addition to frailty, were significantly associated with an increased risk of adverse events (AEs) and prolonged hospital length of stay. Similarly, Aceto et al. [40] reported that the Ariscat (Assess Respiratory Risk in Surgical Patients in Catalonia) score, combined with the modified frailty index (mFI), was predictive of postoperative pulmonary complications in their study.

### Harms caused by AEs

Welch et al. [28] demonstrated that, after adjusting for frailty and age, delirium was associated with increased mortality and longer hospital stays within one month of admission. Similarly, Nowak et al. [35] confirmed that delirium was linked to higher mortality rates, prolonged hospital stays, functional decline and an increased incidence of bleeding among older hospitalised cardiovascular patients.

## Discussion

This systematic review evaluated the relationship between living with frailty and an increased risk of in-hospital AEs among older hospitalised patients. Despite variations in frailty assessment tools and AE definitions across studies, the review identified a relationship between living with frailty and an increased risk of AEs when compared to non-frail. The majority of AEs included delirium, hospital-acquired infections in many forms, falls, pressure ulcers, venous thromboembolism, and several less frequently reported events.

The studies included in this review utilised varying definitions and classifications of AEs, which posed challenges in directly comparing their findings. This variability arose from heterogeneity in study objectives and populations, such as differences between surgical and medical patient cohorts. In addition, the sample sizes varied significantly across studies; while McEvoy et al. [30] included a substantial sample of 44,000 patients, most of the other studies had sample sizes of fewer than 1,000 patients.

Among the 19 included studies, only the study by Joosten et al. [32] did not report a consistent or significant association between frailty and all adverse events examined. While frailty, assessed using the CHS and SOF indices, was a significant predictor of 6-month mortality, its ability to predict in-hospital delirium and falls was limited. This may reflect differences in the sensitivity of the frailty tools used or the relatively small sample size. The authors reported that nearly half of their initial sample dropped out of the study and that most of those who dropped out were frail, which likely reduced statistical power and introduced bias into their results.

Frailty among elderly individuals is a dynamic process characterised by regular transitions between different frailty states [49]. Currently, there is no universally accepted “gold standard” for assessing frailty. Instead, the literature describes a variety of tools, each differing significantly in how they define and measure frailty [13]. This variability was evident in our review, where seven distinct frailty assessment tools were utilised, each with its own classification approach. Among these, the Frailty Index (FI), based on the cumulative deficits model, was the most employed tool. This popularity is likely due to the FI’s reliance on accumulated health deficits, which can be readily extracted from electronic healthcare records or similar datasets [50].

The studies included in this review employed a variety of frailty assessment tools, each with differing conceptual frameworks and levels of complexity. Tools based on the cumulative deficit model—such as the Frailty Index (FI), electronic FI (eFI), modified FI (mFI) and Clegg’s 34-item model—quantify frailty based on the accumulation of health deficits and tend to offer a more granular and continuous measure. In contrast, the Clinical Frailty Scale (CFS) relies on clinical judgement and functional status, making it easier to use in-hospital settings but potentially introducing subjectivity [51]. Conversely, tools like the Edmonton Frail Scale (EFS) and the FRAIL Scale adopt a multidimensional approach, assessing physical health and psychological and social factors. The EFS, for instance, evaluates nine domains, including cognition, general health status, functional independence, social support, medication use, nutrition, mood, continence and functional performance [52]. Similarly, the FRAIL Scale comprises five components: fatigue, resistance, ambulation, illnesses and weight loss, encompassing elements that reflect physical capacity as well as overall health status [53]. On the other hand, the phenotype model focuses on physical frailty characteristics such as weight loss, exhaustion, grip strength, reduced walking speed and reported exhaustion [11]. Another study used a 10-item frailty score adopted from the Veterans Affairs (VA) Cooperative Study health service trial titled “A controlled trial of inpatient and outpatient geriatric evaluation and management” [54], where patients were considered frail if they met two or more of the following conditions: inability to perform one or more basic activities of daily living, stroke within the previous 3 months, history of falls, difficulty walking, malnutrition, dementia, depression, one or more un-planned hospital admissions in the past 3 months, prolonged bed rest, or incontinence.

Limited information was provided on the harms associated with AEs in frail patients, such as prolonged hospital stays, disability or mortality. This lack of harm data may originate from the inherent challenges in establishing clear cause-and-effect relationships within this vulnerable population. It is often difficult to discern whether extended hospital stays or deaths are directly attributable to an AE or are instead a consequence of the underlying illness or surgical recovery for which the patients were hospitalised [8, 22]. It is also challenging to determine whether AEs are solely a result of hospitalisation or if they could have occurred outside healthcare facilities, such as at home. This difficulty arises due to the frailty status of this patient cohort and their advanced age, which contribute to a complex interplay of underlying vulnerabilities, accumulated comorbidities, diverse clinical presentations, poor communication and limited participation in healthcare decision-making and management [22].

### Strength and limitation

This review adopted a comprehensive approach using broad search terms such as “adverse events” alongside specific AEs identified from the literature, including delirium, adverse drug reactions, falls, pressure ulcers, infections and medication errors. These AEs were selected based on their prevalence in published studies and the WHO report *Patient Safety* [4], highlighting common AEs among older hospitalised patients.

A limitation of this review is the limited availability of comprehensive patient safety studies specifically addressing AEs in frail hospitalised patients. Most studies included in this review utilised a mix of definitions and focussed on a limited range of AE types, which reflects the absence of a standardised definition and classification of AEs in the literature. Furthermore, many studies did not report the impact or preventability of AEs in this cohort, which may originate from the lower perceived preventability of AEs in older patients compared to younger patients [8].

The variability in AE definitions, frailty tools and classifications, study aims, populations, and sample sizes made it impossible to conduct a meta-analysis. Such an analysis could have provided more robust conclusions by directly comparing different AE types across varying degrees of frailty. There is a pressing need for more extensive, comprehensive studies encompassing a broader range of AE types and definitions to better understand the incidence and relationship between frailty and AEs during hospitalisation.

## Conclusion

There was an observed association between frailty measured and defined by different frailty tools and concepts and the incidence of AEs, including delirium, hospital-acquired infections (in various forms), hospital falls and pressure ulcers. More comprehensive studies on patient safety incidents among frail hospitalised individuals are needed. These studies should incorporate a broader spectrum of AEs, consider levels of frailty severity, evaluate the impact of comorbidities and explore the influence of gender and race on patient outcomes.

## Supplementary Information

Below is the link to the electronic supplementary material.

Supplementary file1 (DOCX 20 kb)
