# Supplementary material for: The association between frailty and hospital-related adverse events in older hospitalised patients: a systematic literature review
Source: Eur Geriatr Med. 2025 Jun 2;16(4):1303–18. doi: 10.1007/s41999-025-01242-8 (PMC12378845; doi:10.1007/s41999-025-01242-8)
Supplement: Supplementary file 1 — Supplementary file1 (DOCX 20 kb) [file 41999_2025_1242_MOESM1_ESM.docx]

Supplemental Table 1 Search strategy for each database

| MEDLINE = 3637 studies | |
| --- | --- |
| Search | Query |
| #1 | "in hospital*".mp. |
| #2 | hospital*.mp. |
| #3 | "hospital acquired*".mp. |
| #4 | Accidental Falls/ or fall*.mp. |
| #5 | infection*.mp. |
| #6 | Emergence Delirium/ or Delirium/ or delirium*.mp. |
| #7 | "Drug-Related Side Effects and Adverse Reactions"/ or "adverse drug reaction*".mp. |
| #8 | Medication Errors/ or "medication error*".mp. |
| #9 | Pressure Ulcer/ or "Pressure ulcer*".mp. |
| #10 | "Bed Ulcers*".mp. |
| #11 | "Adverse events*".mp. |
| #12 | Frailty/ or Frail Elderly/ or "frail*".mp. |
| #13 | "Hospital-related*".mp. |
| #14 | Inpatient*.mp. |
| #15 | "in-patient*".mp. |
| #16 | Ward*.mp. |
| #17 | unit*.mp. |
| #18 | 1 or 2 or 3 or 13 or 14 or 15 or 16 or 17 |
| #19 | 4 or 5 or 6 or 7 or 8 or 9 or 10 or 11 |
| #20 | 18 and 19 |
| #21 | 12 and 20 |
| Scopus = 4721 studies | |
| Search | Query |
| #1 | (((TITLE-ABS-KEY("infection*")) OR (TITLE-ABS-KEY("fall*")) OR (TITLE-ABS-KEY("delirium*")) OR (TITLE-ABS-KEY("medication error*")) OR (TITLE-ABS-KEY("adverse drug reaction*")) OR (TITLE-ABS-KEY("pressure ulcer*")) OR (TITLE-ABS-KEY("bed ulcer*")) OR (TITLE-ABS-KEY("adverse event*"))) AND ((TITLE-ABS-KEY("hospital acquired*")) OR (TITLE-ABS-KEY("in hospital*")))) AND (TITLE-ABS-KEY("frail*")) |
| CINAHL = 1752 studies | |
| Search | Query |
| #1 | Hospital* |
| #2 | “In hospital*” |
| #3 | “Hospital acquired*” |
| #4 | “Adverse events*” |
| #5 | infection* |
| #6 | Fall* |
| #7 | “Pressure ulcer*” |
| #8 | “Bed ulcer*” |
| #9 | “Adverse drug reaction*” |
| #10 | “Medication errors*” |
| #11 | Delirium* |
| #12 | frail* |
| #13 | S1 OR S2 OR S3 |
| #14 | S4 OR S5 OR S6 OR S7 OR S8 OR S9 OR S10 OR S11 |
| #15 | S13 AND S14 |
| #16 | S12 AND S15 |
| Web of Science = 4810 studies | |
| Search | Query |
| #1 | ALL=(“Adverse events*”) |
| #2 | ALL=(infection*) |
| #3 | ALL=(Fall*) |
| #4 | ALL=(“Pressure ulcer*” ) |
| #5 | ALL=(“Bed Ulcers*”) |
| #6 | ALL=(“Adverse drug reaction*”) |
| #7 | ALL=(“Medication errors*”) |
| #8 | ALL=(Delirium* ) |
| #9 | #1 OR #2 OR #3 OR #4 OR #5 OR #6 OR #7 OR #8 |
| #10 | ALL=(unit*) |
| #11 | ALL=(Ward*) |
| #12 | ALL=("In-patient*") |
| #13 | ALL=(Inpatient*) |
| #14 | ALL=("Hospital-related*") |
| #15 | ALL=("Hospital-acquired*") |
| #16 | ALL=("In-hospital*") |
| #17 | ALL=(Hospital*) |
| #18 | #10 OR #11 OR #12 OR #13 OR #14 OR #15 OR #16 OR #17 |
| #19 | #9 AND #18 |
| #20 | ALL=(frail*) |
| #21 | #19 AND #20 and Article (Document Types) and English (Languages) |

Supplemental Table 2 The modified version of the JBI quality appraisal tool

| Assessment Components | Response options | | | |
| --- | --- | --- | --- | --- |
| 1. Were the aims clearly defined? | Yes | No | Unclear | Not applicable |
| 2. Were the criteria for inclusion in the sample clearly defined? | Yes | No | Unclear | Not applicable |
| 3. Were the study subjects and the setting described in detail? | Yes | No | Unclear | Not applicable |
| 4. Were the adverse events adverse event identified in a valid and reliable way? | Yes | No | Unclear | Not applicable |
| 5. Were the included sample clearly tested for the presence of the adverse event prior to or at admission? | Yes | No | Unclear | Not applicable |
| 6. Were frailty defined and measured using a clear and reliable tool? | Yes | No | Unclear | Not applicable |
| 7. Were the outcomes measured in a valid and reliable way? | Yes | No | Unclear | Not applicable |
| 8. Were confounding factors identified? | Yes | No | Unclear | Not applicable |
| 9. Were strategies to deal with confounding factors stated? | Yes | No | Unclear | Not applicable |
| 10. Was an appropriate statistical analysis used? | Yes | No | Unclear | Not applicable |
| Overall appraisal: Include □ Exclude □ Seek further info □ | | | | |

Supplemental Table 3 Further characteristics of the included studies

| Authors | Aim | Method | Sample age (years) | Frailty and AE association | Authors conclusion |
| --- | --- | --- | --- | --- | --- |
| Deiner S et al [50] | To examine the relationship between various frailty measures and the incidence and severity of delirium | prospective observational study | 70+ | The frailty index was linked to an increased incidence of delirium | Both a frailty index and a frailty phenotype measure were associated with postoperative delirium, though the index demonstrated a stronger, adjusted relationship. Preoperative frailty assessment may therefore be useful in identifying patients at higher risk. |
| Esmaeeli S et al [38] | To assess the association between preoperative frailty and the occurrence of postoperative delirium (POD) in elderly orthopaedic trauma patients | retrospective observational study | 65+ | Multivariable regression showed each unit increase in the FRAIL score raised the likelihood of POD by 33%. | preoperative frailty substantially increases the likelihood of postoperative delirium (POD) among hospitalised older adults with orthopaedic trauma. |
| Sieber F et al [40] | To evaluate the relationship between vulnerability assessed using the Edmonton Frailty Scale (EFS) and postoperative delirium (POD) in older non-ICU patients undergoing low-risk elective surgery | retrospective Observational study | 65+ | EFS ≥6 was consistently linked to postoperative delirium in all bivariate models | an EFS score of 6 or higher emerged as a strong predictor of postoperative delirium in older elective surgical patients who did not require ICU admission |
| Joseph B et al [36] | To quantify the prevalence of Frailty Syndrome (FS) in geriatric trauma patients and examine its association with trauma readmissions, recurrent falls, and 6-month mortality. | prospective observational study | 65+ | Frail patients had an increased likelihood of developing in-hospital complications | TSFI is a valuable tool for detecting older trauma patients at risk of both short- and long-term outcomes, underlining the importance of early, targeted interventions. |
| Leung J et al [51] | To assess whether preoperative frailty independently predicts the risk of postoperative delirium | retrospective Observational Study | 65+ | Preoperative frailty was independently linked to postoperative delirium | frailty assessment in the preoperative setting improves risk evaluation, particularly in identifying older patients at risk of postoperative delirium |
| Hanlon J et al [34] | To evaluate the prevalence and identify predictors of inappropriate prescribing in hospitalised frail elderly patients | retrospective Observational Study | 65+ | Frailty is linked to inappropriate medication prescribing and use in older hospitalised veterans | inappropriate drug prescribing is common among frail elderly veteran inpatients, largely influenced by polypharmacy and underlying health conditions. |
| McEvoy L et al [41] | To explore the relationship between increasing frailty in older surgical patients and the risk of specific hospital-acquired AEs | Retrospective observational study | 70+ | Increasing frailty was linked to a higher risk of adverse hospital events, with the risk rising as the number of frailty deficit items increased. Patients in the highest deficit group (4–12 items) faced greater risks compared to those in the lowest group (0–1 item) | Frailty increases the risk of adverse events in older surgical patients during hospitalisation. |
| Thillainadesan J et al [37] | To examine the association between frailty and hospital-acquired geriatric syndromes in older hospitalised vascular surgery patients, and to assess the prognostic accuracy of the Frailty Index (FI) and Clinical Frailty Scale (CFS) in predicting delirium and functional decline | prospective observational study | 65+ | Frailty status is strongly associated with a higher risk of hospital-acquired geriatric syndromes and delirium in older patients admitted to vascular surgery | Frail older vascular surgery patients are at higher risk of hospital-acquired geriatric syndromes. The FI and CFS are effective tools for predicting delirium, although not all frail patients experience it. |
| Hubbard R et al [42] | To assess the predictive validity of the FI-AC and its association with various adverse outcomes in older inpatients | Prospective observational study | 70+ | The Frailty Index (FI) was significantly associated with an increased likelihood of in-hospital falls, delirium, pressure ulcer incidence and functional decline. | A higher FI-AC score was significantly linked to multiple adverse outcomes. |
| Chan S et al [46] | To examine the association between the Clinical Frailty Scale (CFS) and in-hospital complications as well as length of stay | retrospective observational study | 65+ | Frailty was a predictor of increased mortality, longer length of stay, and higher postoperative complications | Preadmission frailty, assessed using the CFS, is associated with discharge destination, in-hospital complications, and length of stay. |
| Dasgupta M et al [49] | To investigate the relationship between frailty and the increased risk of postoperative complications in older adults with medical conditions undergoing major non-cardiac elective surgery | Retrospective observation study (blinded char review) | 70+ | Increasing frailty was linked to postoperative complications, longer hospital stays, and difficulty being discharged home | Frailty screening enhances risk prediction for postoperative complications in older adults undergoing elective non-cardiac surgery. |
| Jung H et al [44] | To evaluate the effectiveness of the at-point Clinical Frailty Scale (CFS) in predicting clinical outcomes for older inpatients | prospective Observational Study | 65+ | At-point CFS can predict falls, pressure ulcers, and delirium | At-point CFS assessment in older inpatients identifies high-risk individuals prone to adverse geriatric conditions and poor hospital outcomes.  and negative. |
| Kim D et al [35] | To assess the influence of preoperative central sarcopenia, frailty, and comorbidities on surgical outcomes in elderly patients with degenerative spinal diseases (DSD) | Retrospective observation study | 65+ | Postoperative complications were significantly associated with both surgical invasiveness and the K-FRAIL scale | Frailty, comorbidity, and surgical invasiveness are key risk factors for postoperative complications and length of stay in elderly patients with DSD. Preoperative identification of these factors supports perioperative optimisation, risk stratification, and patient counselling. |
| Nowak W et al [45] | To evaluate the impact of frailty on the risk of complications in patients aged 65 years and older hospitalised for acute coronary syndrome (ACS) | prospective Observational Study | 65+ | Frailty was linked to multiple in-hospital complications in patients aged ≥65 years with a diagnosis of acute coronary syndrome (ACS) | Frailty syndrome significantly increases in-hospital complications in patients aged ≥65 years with acute coronary syndrome (ACS). |
| Chen D et al [48] | To explore the relationship between frailty and postoperative pulmonary complications (PPCs) in elderly patients undergoing video-assisted thoracoscopic pulmonary resections, and to assess the added value of frailty evaluation in enhancing the predictive accuracy of the PPC risk index and ASA classification | prospective observational study | 65+ | Preoperative frailty was associated with postoperative pulmonary complications (PPCs) in elderly patients undergoing video-assisted thoracoscopic pulmonary resections. | Frailty is associated with postoperative pulmonary complications (PPCs) in elderly patients undergoing video-assisted thoracoscopic pulmonary resections. Including frailty assessment improves the predictive accuracy of the PPC risk index and ASA classification. |
| P. Aceto et al [33] | To assess the effectiveness of the modified Frailty Index (mFI) in predicting postoperative pulmonary complications (PPCs) in elderly patients undergoing major open abdominal surgery. | prospective observational study | 65+ | Frail patients (mFI ≥0.18) were at higher risk for postoperative pulmonary complications (PPCs) | Elderly patients with an mFI ≥0.18 and/or an Ariscat score ≥27 are at increased risk of postoperative pulmonary complications (PPCs) following open major abdominal surgery. |
| Welch C et al [39] | To evaluate patient and hospital factors, including frailty measures, that predict delirium, its screening, and recognition | prospective observational study | 65+ | The presence of delirium was associated with higher Clinical Frailty Scale (CFS) scores | Frailty is strongly linked to the development of delirium, though it is less likely to be recognised in frail patients. |
| Birkelbach O et al [47] | To investigate the relationship between frailty status and the occurrence of in-hospital postoperative complications in elderly surgical patients across various surgical specialties | Retrospective observational analysis | 65+ | Phenotypic pre-frailty and frailty were strongly associated with an increased risk of postoperative complications | Fried’s frailty phenotype is an effective predictor of in-hospital postoperative complications across surgical specialties and can be seamlessly integrated into clinical practice. Both pre-frailty and frailty, regardless of age, help identify at-risk patients and inform patient counselling, care planning, and risk mitigation strategies. |
| Joosten E et al [43] | To evaluate the extent to which frailty predicts delirium and falls during hospitalisation | prospective Observational Study | 70+ | Frailty, as identified by the CHS and SOF indexes, was not found to be a risk factor for delirium or falls | Frailty, assessed using the CHS index, is an independent risk factor for 6-month mortality. However, both the CHS and SOF indexes have limited utility in evaluating specific geriatric outcomes like falls and delirium in hospitalised older patients. |
